# The roles of stem cell memory T cells in hematological malignancies

**DOI:** 10.1186/s13045-015-0214-5

**Published:** 2015-10-14

**Authors:** Ling Xu, Yikai Zhang, Gengxin Luo, Yangqiu Li

**Affiliations:** Department of Hematology, First Affiliated Hospital, Jinan University, Guangzhou, 510632 China; Institute of Hematology, Jinan University, Guangzhou, 510632 China; Key Laboratory for Regenerative Medicine of Ministry of Education, Jinan University, Guangzhou, 510632 China

**Keywords:** Stem cell memory T cells, Adoptive cell therapy, Hematological malignancy, Antigen-specific T cells

## Abstract

Adoptive cell therapy (ACT) is rapidly migrating from bench to clinical therapy for hematological malignancies. Recently, a new subtype of memory T cells, stem cell memory T (T_SCM_) cells, was shown to be one of the most favorable subsets for ACT. T_SCM_ has high self-renewal capacity and is associated with superior T cell engraftment, persistence, and antitumor immunity. In this review, we focused on the characteristics of antigen-specific T_SCM_ cells and discussed their potential for immunotherapy targeting hematological malignancies.

## Introduction

T cell immunodeficiencies have been observed in patients with hematological disorders [1]. These deficiencies lead to the expansion of malignant clones and are thought to play an important role in tumorigenesis [2–5]. To design an effective approach for recovering T cell immunity, particularly antigen-specific T cell immunity, it is necessary to accurately evaluate the T cell immune status at either the molecular or cellular level, including characteristics such as recent thymic output function, number of naive T cells, diversity in the T cell receptor (TCR) repertoire, and tumor antigen-specific cytotoxicity T cell clones [6–9]. More recently, stem cell memory T (T_SCM_) cells have been described as a new immune biomarker for evaluating long-term memory T cell immune reconstitution, which is an important index after hematopoietic stem cell transplantation (HSCT) [10–12]. T_SCM_ cells have been shown to be able to differentiate into central memory T cells (T_CM_), effector memory (T_EM_), and terminal effector T cells (T_TE_).

Adaptive immunity is characterized by the ability to form long-lived immunological memory. Memory T cells develop when antigen-specific naive CD4^+^ or CD8^+^ T cells become activated upon antigen exposure and subsequently undergo proliferative expansion and differentiation [13, 14]. Therefore, efficient and persistent immune memory is essential for long-term protection against infections and malignancies. Memory T cells play a critical role in maintaining this immune defense [15]. T_SCM_ cells are considered as an important immune marker for the repopulating T cell pool and immune reconstitution which is associated with favorable clinical outcome after HSCT [16]. T_SCM_ cell research may support the advances in biomarker research, diagnosis, and therapy for hematological malignancies [17–20]. Moreover, T_SCM_ cell research may be important for understanding and influencing diverse chronic immune reactions, including graft-versus-host disease (GVHD) [21].

## T_SCM_ cell characteristics

**Fig. 1:**
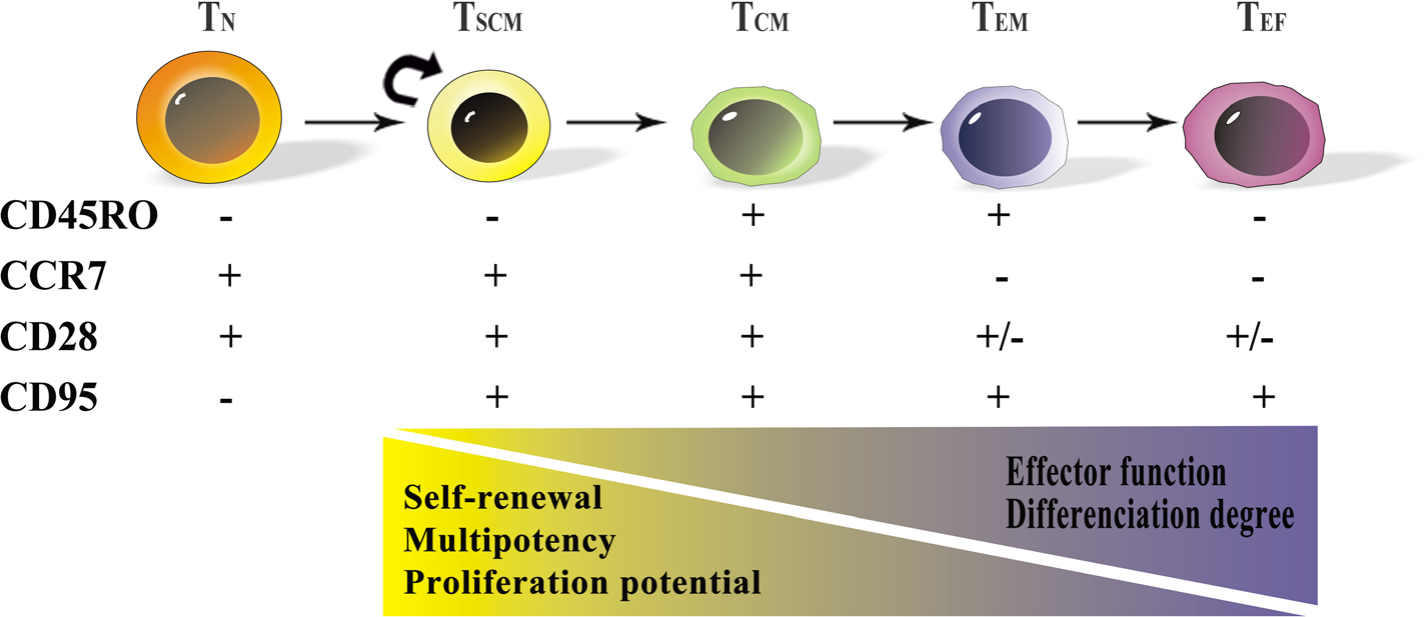
Schematic model for T cell differentiation. Upon activation, naive T cells differentiate into various memory and effector cells. Self-renewal capacity, multipotency, and proliferation potential decrease upon differentiation. The expression of CD45R0, CCR7, CD28, and CD95 markers changes during T cell differentiation from T_N_ to T_TE._ The minimum set of canonical markers can be used to identify the five major subsets of T cells. *T*_*N*_ naive T cell, *T*_*SCM*_ stem cell memory T cell, *T*_*CM*_ central memory T cell, *T*_*EM*_ effector memory T cell, *T*_*TE*_ terminal effector T cell

Memory T cells (including CD4^+^ and CD8^+^ memory T cells) include several subtypes: stem cell memory (T_SCM_), central memory (T_CM_), transitional memory (T_TM_) (described only in CD4^+^ memory T cells), effector memory (T_EM_), and terminal effector (T_TE_) T cells [16, 22]. T_SCM_ cells were first observed in a murine model of GVHD by Zhang et al., who reported a new subset of post-mitotic CD44^lo^CD62L^hi^CD8^+^ T cells expressing Sca-1 (stem cell antigen 1), CD122, and Bcl-2. This population of T cells was able to generate and sustain all allogeneic T cell subsets in GVHD reactions. These alloreactive CD8^+^ T cells were demonstrated to have enhanced self-renewal capacity and multipotency. These cells are capable of differentiating into T_CM_, T_EM_, and T_TE_ cells [14, 21]. In humans, an example came from the identification of a population of naive yellow fever (YF)-specific CD8^+^ T cells after vaccination. These cells were stably maintained for more than 25 years and were capable of ex vivo self-renewal. In-depth analysis of markers and genome-wide mRNA profiling have shown that these cells are distinct from genuine naive cells from unvaccinated donors and resemble the recently described stem cell-like memory subset T_SCM_ [23]. Moreover, epigenetic analysis has also revealed that histone modifications and gene expression signatures could distinguish T_SCM_ from other CD8^+^ T cell subsets [24]. Increasing data have supported the notion that the human T_SCM_ subset is a clearly distinct subset in between the naive T cell (T_N_) and T_CM_ subsets. Human T_SCM_ cells have been described as a long-lived memory T cell population which are CD45RO^−^, CCR7^+^, CD45RA^+^, CD62L^+^, CD27^+^, CD28^+^, and IL-7Rα^+^. These markers are characteristic of naive T cells. The immunophenotypic markers expressed in different T cell subtypes (from T_N_ to T_TE_ cells) are summarized in Table 1. T_SCM_ cells express increased levels of CD95, IL-2Rβ, CXCR3, and LFA-1 and exhibit numerous functional attributes distinct from memory cells. However, human T_SCM_ cells constitute only approximately 2–4 % of the total CD4^+^ and CD8^+^ T cell population in the periphery and can be identified by polychromatic flow cytometry based on the simultaneous expression of several naive markers together with the memory marker CD95 [25]. A linear T cell differentiation model and the minimum set of markers used for identifying and sorting T_SCM_ are depicted in Fig. 1 [25].

**Table 1:** Summary of the expression of functional molecules in circulating naive, memory T cell and terminal effector T cell subsets

Subset	Phenotype
T_N_	CD45RO^−^CCR7^+^CD45RA^+^CD62L^+^CD27^+^CD28^+^CD127^+^(IL-7Rα^+^)CD95^−^CD103^−^
T_SCM_	CD45RO^−^CCR7^+^CD45RA^+^CD62L^+^CD27^+^CD28^+^CD127^+^(IL-7Rα^+^)CD95^+^CD103^−^
T_CM_	CD45RO^+^CCR7^+^CD45RA^−^CD62L^+^CD28^+^CD27^+^CD127^+^(IL-7Rα^+^)CD95^+^CD103^−^
T_EM_	CD45RO^+^CCR7^−^CD45RA^−^CD62L^−^CD28^−/+^CD27^−/+^CD127^−/+^(IL-7Rα^−/+^)CD95^+^CD103^+^
T_TE_	CD45RO^−^CCR7^−^CD45RA^+^CD62L^−^CD28^−/+^CD27^−^CD127^−^(IL-7Rα^−^)CD95^+^CD103^−^

“*+*” positive expression, “*−*” negative expression, *T*_*N*_ naive T cell, *T*_*SCM*_ stem cell memory T cell, *T*_*CM*_ central memory T cell, *T*_*EM*_ effector memory T cell, *T*_*TE*_ terminal effector T cell

Self-renewing memory T cells may be regulated by shared signaling pathways such as those involved in hematopoietic stem cells or memory B cells. The Wnt-β-catenin pathway is an evolutionarily conserved pathway that regulates hematopoietic stem cell self-renewal and multipotency by limiting stem cell proliferation and differentiation. Similarly, a key role for Wnt signaling during the maintenance of “stemness” in CD8^+^ T_SCM_ cells was demonstrated by Gattinoni et al. It was shown that disrupting the Wnt/β-catenin pathway by glycogen synthase-3β (GSK-3β) inhibitors promoted the generation of CD44^low^CD62L^high^Sca-1^high^CD122^high^Bcl-2^high^ self-renewing multipotent CD8^+^ T_SCM_ cells with proliferative and antitumor capacities that exceeded those of the T_CM_ and T_EM_ subsets [11, 26, 27]. In addition, antigen-specific T_SCM_ cells were shown to preferentially reside in the lymph nodes (LNs) and less so in the spleen and bone marrow [28].

There are numerous factors that act as modulators regulating the maturation and activation of CD8^+^ T cells, for example, suppressor of cytokine signaling (SOCS) is one of the key modulators [29]. Moreover, it has been reported that activation of naive T cells with anti-CD3 and anti-CD28 antibody-conjugated beads in the presence of low doses of IL-7 and IL-15 promotes the generation of CD45RA^+^CD62L^+^CCR7^+^CD95^+^ T_SCM_ cells [30].

## Antigen-specific T_SCM_

It is well known that antigen-specific T cells are crucial components for antitumor or antivirus immunity in patients with hematological malignancies, particularly in patients after HSCT. It is possible that the number of antigen-specific T_SCM_ cells may be the determining factor of immunity. However, there have been few reports on antigen-specific T_SCM_ cells. Low frequency of these cells limits detailed characterization. For example, <1 % of total human T cells are defined as CD8^+^CD45RA^+^CCR7^+^CD127^+^CD95^+^ viral-specific T_SCM_ cells. Human CMV-specific T_SCM_ cells can be detected at frequencies similar to those observed in other subsets, with frequency around ∼1/10,000 T cells [31, 32].

Antigen-specific T_SCM_ cells represent a long-lasting component of the cellular immune response to viruses and tumor-associated antigens (TAAs). For virus-specific T_SCM_ cells, research has first focused on human immunodeficiency virus type 1 (HIV-1)-specific CD8^+^ T_SCM_ cells. It is known that HIV-specific CD8^+^ T cells can influence HIV-1 disease progression during untreated HIV-1 infections, and recent data have shown that HIV-1-specific CD8^+^ T_SCM_ cells are detectable in all stages of HIV-1 infection. These cells were found to be increased in number in patients receiving suppressive antiretroviral therapy when compared with those untreated patients [33]. It was found that CD4^+^ T_SCM_ cells were susceptible to HIV infection; thus, HIV-1 virus may exploit the stem cell characteristics of cellular immune memory T cells and lead to long-term viral persistence [34]. Similar findings were demonstrated in a study of human T cell leukemia virus type 1 (HTLV-1)-infected CD4^+^ T_SCM_ cells in patients with adult T cell leukemia (ATL). This report first demonstrated an association between T cell malignancy and T_SCM_ cells. T_SCM_ cells from ATL patients were capable of sustaining themselves in a less proliferative mode, yet they were able to differentiate into other memory T cell populations during the rapidly propagating phase. These cells have been identified at the hierarchical apex capable of reconstituting identical ATL clones [35]. A decrease in the infection of CD4^+^ T_SCM_ cells was found to preserve CD4^+^ T cell homeostasis and prevents disease progression despite significant viremia in both HIV-1 and HTLV-1 infections [36].

T_SCM_ cells may play a major role in specific antitumor response and long-term immune surveillance directed against tumors [17, 37, 38]. In addition, T_SCM_ cells have been proposed to be one of the key determinants of immune memory. It may be interesting to monitor the level of T_SCM_ cells and its significance for immune reconstitution and prognosis of patients with hematological malignancies before and after therapy, particularly HSCT. There have been only a few studies on TAA-specific T_SCM_ cells. Recently, dynamic changes of T_SCM_ cells were longitudinally tracked in patients who underwent haploidentical HSCT. These studies demonstrated that donor-derived T_SCM_ cells were highly enriched early after HSCT. T_SCM_ cells can differentiate directly from naive precursors infused in the grafts. Through T cell receptor (TCR) gene analysis, T_SCM_ cells have been found to have diversification in immune memory after allogeneic HSCT [10]. It was also demonstrated that the level of T_SCM_ cells may be used to evaluate immune reconstitution in patients who received posttransplant cyclophosphamide (pt-Cy) for GVHD prophylaxis. Similarly, donor-derived T_SCM_ cells were found to be the most abundant circulating T cell population in the early days following haploidentical HSCT and pt-Cy. These donor-derived T_SCM_ cells preceded the expansion of effector cells. Antigen-specific T_SCM_ cells generated detectable recall responses; thus, it has been proposed to explore T_SCM_ cells derived from donor naive precursor cells in the clinical setting to overcome immunodeficiency [12]. With the ability to expand and differentiate into effectors capable of mediating potent xenogeneic GVHD in immunodeficient mice, these donor naive precursor-derived T_SCM_ cells were noted to be superior to other memory lymphocytes. Furthermore, gene-modified T_SCM_ cells were found to be the only T cell subset capable of expanding and mediating GVHD in serial transplantations [30]. These findings indicate negative aspects of T_SCM_ cells for clinical application.

## The potential of T_SCM_ cells in immunotherapy for hematological malignancies

T_SCM_ cells may be a novel and critical therapeutic resource because these cells have the potential to serve as a stable cellular vehicle. Two gene therapy clinical trials with gene-corrected hematopoietic stem cells provided a glimpse into this possibility. Long-term in vivo T cell reconstitution was characterized in these trials. Specifically, the investigators traced the fate of greater than 1700 individual T cell clones in patients who underwent gene therapy. The studies demonstrated that the T_SCM_ cells persisted and preserved their precursor potential in humans for up to 12 years after the infusion of gene-corrected stem cells [39]. The demonstration of the safe, functional, and decade-long survival of the engineered T_SCM_ cells in humans sets the stage for their clinical application. Since T_SCM_ cells were shown to be capable of reconstituting the full repertoire of memory and effector T cells after HSCT, it is particularly attractive to use them for adoptive immunotherapies. T_SCM_ cells might overcome current limitations, such as inefficient T cell engraftment, poor persistence, and inability to mediate prolonged immune attacks [10–12, 40].

Even though potent antitumor activity of T_SCM_ cells was demonstrated in preclinical animal tumor models [26, 27], it is currently not feasible to treat patients with naturally occurring T_SCM_ cells because it is a scarce and small proportion of circulating lymphocytes. Therefore, strategies that can generate, expand, and enable the redirection of T_SCM_ cells against cancer cells need to be defined. Cieri and colleagues have recently described that a large number of T_SCM_ cells were generated by priming T cells with low doses of IL-7 and IL-15. It is therefore possible to generate, expand, and genetically engineer T_SCM_ cells in vitro from naive precursors. Furthermore, the in vitro-generated T_SCM_ cells displayed enhanced proliferative capacity upon adoptive transfer into immunodeficient mice, a finding consistent with those using naturally occurring T_SCM_ cells [11, 30]. T_SCM_ cells were also expanded from naive precursors by inhibiting Akt signaling during ex vivo priming and expansion. The Akt-inhibited minor histocompatibility antigen (MiHA)-specific CD8^+^ T cells had superior expansion capacity in vitro and induced superior antitumor activity in multiple myeloma-bearing immunodeficient mice. These findings provided a rationale for clinically exploiting ex vivo-generated, Akt-inhibited, MiHA-specific CD8^+^ T cells or TAA-specific CD8^+^ T cells for adoptive immunotherapy [41, 42]. Schmueck-Henneresse et al. also described a simplified culture protocol allowing for fast expansion of virus-specific T_SCM_ cells from a mixed T_N_/T_SCM_ pool of peripheral blood lymphocytes. This may be the basis for novel cell therapeutic options for life-threatening viral infections [31]. Among the known memory T cell subpopulations, the T_SCM_ cell subset has profound implications for the design and development of effective vaccines as well as T cell-based therapies [13, 26, 28]. As immunotherapy plays increasingly important roles in cancer management, further exploration of T_SCM_ cells and their regulation may facilitate clinical development of humoral (monoclonal antibodies and inhibitors of B cell receptor signaling) and cellular (CART) immunotherapies [40, 43–47].

## Conclusions and future perspectives

T_SCM_ cells have the capacities of self-renewal and differentiation into various memory/effector subsets. These cells can lead to superior immune reconstitution. The identification of human T_SCM_ cells is directly relevant for evaluating life-long cellular immune status, immune reconstitution after allogeneic HSCT, and design of vaccines and T cell immunotherapy. However, it remains unclear at this time whether the number of T_SCM_ cells may be used as a standard biomarker for immune reconstitution after HSCT. In addition, the low number of T_SCM_ cells in circulating lymphocytes is also limiting their application [11]. Strategies for in vitro and in vivo isolation and generation of highly effective antitumor T_SCM_ cells are under intensive investigation.

## Abbreviations

ACT: adoptive cell therapy
ATL: adult T cell leukemia
HTLV-1: human-cell leukemia virus type 1
MiHAs: minor histocompatibility antigens
TAA-CTL: tumor antigen associated cytotoxic T lymphocytes
T_CM_: central memory T cell
TCR: T cell receptor
T_EM_: effector memory T cell
T_N_: naive T cell
T_SCM_: stem cell memory T cell
T_TE_: terminal effector T cell
T_TM_: transitional memory T cell
